# Red cell distribution width as a predictor for bronchopulmonary dysplasia in premature infants

**DOI:** 10.1038/s41598-021-86752-8

**Published:** 2021-03-31

**Authors:** Hayato Go, Hitoshi Ohto, Kenneth E. Nollet, Kenichi Sato, Hirotaka Ichikawa, Yohei Kume, Yuji Kanai, Hajime Maeda, Nozomi Kashiwabara, Kei Ogasawara, Maki Sato, Koichi Hashimoto, Mitsuaki Hosoya

**Affiliations:** 1 Department of Pediatrics, Fukushima Medical University School of Medicine, Hikarigaoka 1, Fukushima, Japan; 2 Fukushima Medical University, Fukushima, Japan; 3 Department of Blood Transfusion and Transplantation Immunology, Fukushima Medical University School of Medicine, Fukushima, Japan

**Keywords:** Biomarkers, Medical research

## Abstract

Bronchopulmonary dysplasia (BPD) is the most common morbidity complicating preterm birth. Red blood cell distribution width (RDW), a measure of the variation of red blood cell size, could reflect oxidative stress and chronic inflammation in many diseases such as cardiovascular, pulmonary, and other diseases. The objectives of the present study were to evaluate perinatal factors affecting RDW and to validate whether RDW could be a potential biomarker for BPD. A total of 176 preterm infants born at < 30 weeks were included in this study. They were categorized into BPD (n = 85) and non-BPD (n = 91) infants. RDW at birth and 14 days and 28 days of life (DOL 14, DOL 28) were measured. Clinical data were obtained from all subjects at Fukushima Medical University (Fukushima, Japan). The mean RDW at birth, DOL 14 and DOL 28 were 16.1%, 18.6%, 20.1%, respectively. Small for gestational age (SGA), chorioamnionitis (CAM), hypertensive disorders of pregnancy (HDP), gestational age and birth weight were significantly associated with RDW at birth. SGA, BPD and red blood cell (RBC) transfusion before DOL 14 were associated with RDW at DOL 14. BPD and RBC transfusion before DOL 14 were associated with RDW at DOL 28. Compared with non-BPD infants, mean RDW at DOL 14 (21.1% vs. 17.6%, *P* < 0.001) and DOL 28 (22.2% vs. 18.2%, *P* < 0.001) were significantly higher in BPD infants. Multivariate analysis revealed that RDW at DOL 28 was significantly higher in BPD infants (*P* = 0.001, odds ratio 1.63; 95% CI 1.22–2.19). Receiver operating characteristic analysis for RDW at DOL 28 in infants with and without BPD yielded an area under the curve of 0.87 (95% CI 0.78–0.91, *P* < 0.001). RDW at DOL 28 with mild BPD (18.1% vs. 21.3%, *P* < 0.001), moderate BPD (18.1% vs. 21.2%, *P* < 0.001), and severe BPD (18.1% vs. 24.0%, *P* < 0.001) were significantly higher than those with non-BPD, respectively. Furthermore, there are significant differences of RDW at DOL 28 among mild, moderate, and severe BPD. In summary, we conclude that RDW at DOL 28 could serve as a biomarker for predicting BPD and its severity. The mechanism by which RDW at DOL 28 is associated with the pathogenesis of BPD needs further elucidation.

## Introduction

Bronchopulmonary dysplasia (BPD) is the leading cause of chronic lung disease with long term respiratory and neurodevelopmental outcomes^1,2^. It is associated with maternal inflammation, surfactant deficiency, mechanical ventilation and oxygen toxicity that provokes an inflammatory response and impairs lung development with dysregulated angiogenesis and alveolarization^3,4^. Premature infants are often exposed to positive pressure ventilation and supplemental oxygen, contributing to the development of BPD, which is characterized by an arrest of vascular and alveolar growth and high risk for pulmonary hypertension^4^. Such structural alterations are accompanied by characteristic inflammatory changes, oxidative stress and endothelial dysfunction^5^. While the etiology of BPD is multifactorial, the relevance of oxidative stress and chronic inflammation to lung injury in premature infants has been demonstrated in human subjects and animal models exposed to hyperoxia^6,7^.

Red blood cell distribution width (RDW), a measure of the variation red blood cell size, is a routinely and rapidly reported component of an automated complete blood cell count (CBC). Typically, RDW has been used to differentiate causes of anemia, but recent research has suggested that RDW could reflect oxidative stress and chronic inflammation in many diseases such as heart failure, chronic obstructive pulmonary disease, pulmonary hypertension, idiopathic pulmonary fibrosis, and cancer^8–11^. Furthermore, an adult population-based study in the U.S has observed an inverse relationship between RDW and lung function^12^. Although RDW in adults is known to correlate with lung disease, cardiovascular disease, and cancer, no reports have investigated whether RDW during the postnatal period could be a predictor for neonatal lung disease such as BPD in a large cohort.

Therefore, in this study, we hypothesized that postnatal RDW could serve as a biomarker of BPD. The objectives of the present study were to evaluate the perinatal factors affecting RDW and to validate its biomarker potential for BPD.

## Results

### Clinical characteristics and RDW in preterm infants.

**Figure 1:**
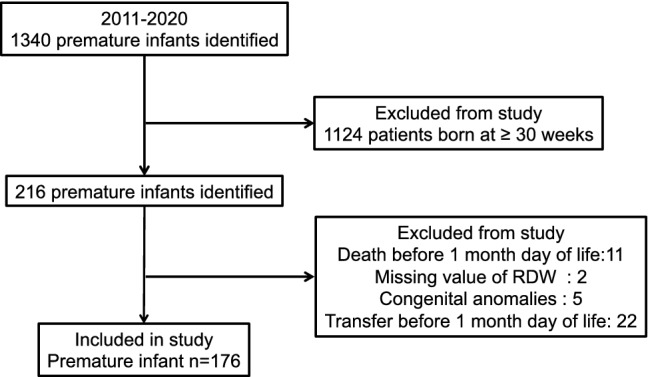
Flow chart of this study.

**Figure 2:**
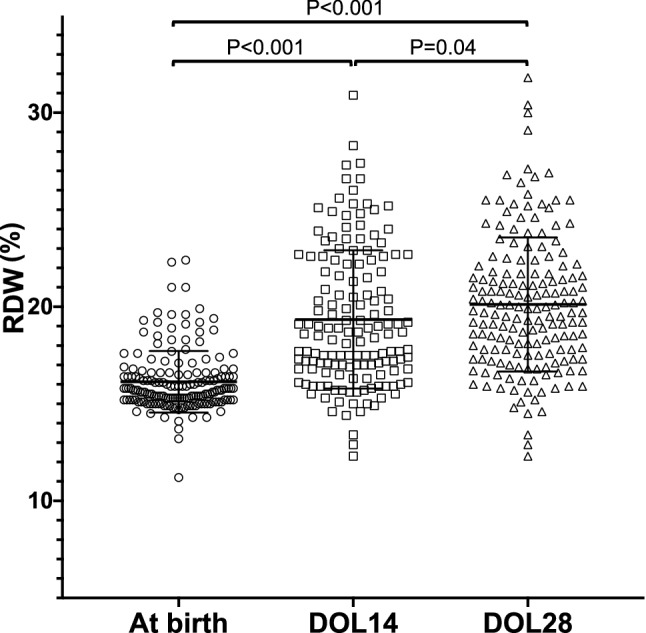
Postnatal red cell distribution width (RDW) in all subjects (n = 176). The *x*-axis represents days of life (DOL), and *y*-axis is RDW (%). DOL 14: 14 days of life, DOL 28: 28 days of life. Horizontal bars denote the mean ± SD in each group of infants. The P values were calculated using Related-Samples Friedman's Two-Way Analysis of Variance by Ranks.

**Figure 3:**
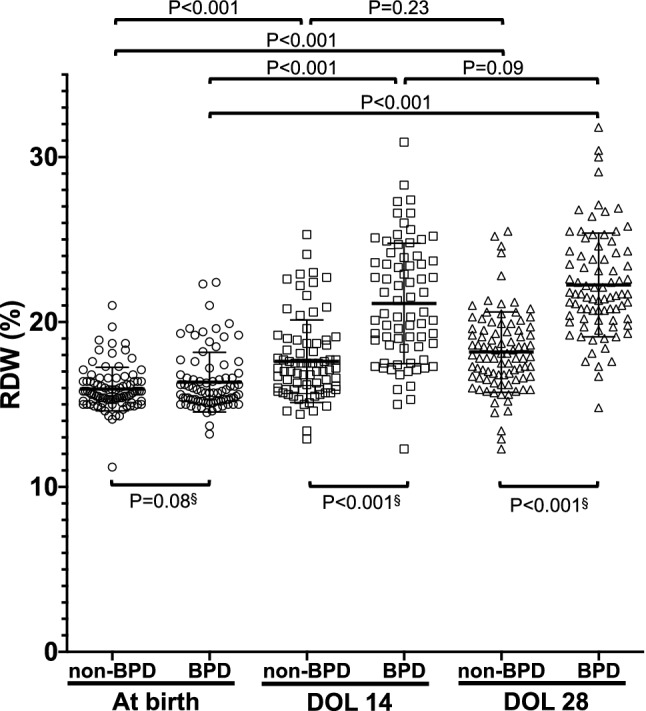
Postnatal red cell distribution width (RDW) in non-BPD (n = 90) and BPD (n = 82) infants. DOL 14: 14 days of life, DOL 28: 28 days of life. Horizontal bars denote the mean ± SD in each group of infants. The P values were calculated using Related-Samples Friedman's Two-Way Analysis of Variance by Ranks unless otherwise noted. §: Student’s t-test. *BPD* bronchopulmonary dysplasia. *DOL* days of life.

As shown in Fig. 1, a total of 176 preterm (BPD: n = 85, non-BPD: n = 91) infants were included in this study. The clinical characteristics of those born at less than 30 weeks are summarized in Table 1. The mean (± SD) GA and BW were 26.1 (± 0.1) weeks and 798 (± 19) grams, respectively. Regarding BPD, 29 premature infants were diagnosed with mild BPD, 27 with moderate BPD, 29 with severe BPD, and 91 had no BPD. Table 2 shows the factors affecting RDW at birth, DOL 14 and DOL 28, respectively. SGA, HDP, CAM, gestational age and birth weight were significantly associated with RDW at birth. As shown in supplemental Figure S1, RDW at birth was positively but weakly correlated with gestational age (*P* = 0.04, *r* = 0.15) and negatively but weakly correlated with birth weight (*P* < 0.001, *r* = *−* 0.26). In terms of RDW at DOL 14, SGA and red blood cell transfusion before DOL 14 were significantly associated. BPD was also significantly associated with RDW at DOL 14 and 28 (Table2). The mean RDW at birth, DOL 14 and DOL 28 were 16.1%, 18.6%, 20.1%, respectively. Interestingly, RDW gradually increased during the postnatal period (Fig. 2). Table 3 shows a comparison of the clinical characteristics and RDW among groups with and without BPD. The mean gestational age and birth weight in BPD neonates were significantly lower than that in non-BPD infants (24.9 vs. 26.1 weeks, *P* < 0.001, 646 g vs. 937 g, *P* < 0.001), respectively. Multivariate analysis revealed that in BPD infants, duration of oxygen supplementation in BPD infants was significantly longer than in non-BPD infants (*P* = 0.001, odds ratio 1.16; 95% CI 1.06–1.27). Although RDW at birth and DOL 14 were not significantly higher in BPD infants, RDW at DOL 28 was significantly higher in BPD infants (*P* = 0.001, odds ratio 1.63; 95% CI 1.22–2.19) (Table 3). As shown Fig. 3, compared with non-BPD infants, mean RDW at DOL 14 (21.1% vs. 17.6%, *P* < 0.001), and DOL 28 (22.2% vs. 18.2%, *P* < 0.001) were higher in BPD infants.

**Table 1:** Characteristics of subjects.

	Total (N = 176)	
Gestational age, mean ± SD, weeks	26.1	± 0.1
Birth weight, mean ± SD, grams	798	± 19
Male phenotype, n (%)	87	(49.4)
CAM, n (%)	81	(46.0)
Antenatal steroid, n (%)	136	(77.2)
PROM, n (%)	38	(21.6)
HDP, n (%)	21	(11.8)
RDS, n (%)	145	(82.4)
SGA, n (%)	28	(15.9)
PDA, n (%)	91	(51.1)
Apgar score at 1 min median (IQR)	4	(2–5)
Apgar score at 5 min median (IQR)	7	(5–8)
BPD, n (%)	85	(48.3)
Mild BPD	29	(16.4)
Moderate BPD	27	(15.3)
Severe BPD	29	(16.4)
Inhaled nitric oxide	19	(10.7)
Duration of oxygen supplementation mean ± SD, week	13.1	± 8.7

*IQR* interquartile range, *CAM* chorioamnionitis, *PROM* premature rupture of membranes, *HDP* hypertensive disorders of pregnancy, *SGA* small for gestational age, *RDS* respiratory distress syndrome, *PDA* patent ductus arteriosus, *BPD* bronchopulmonary dysplasia, *DOL* days of life.

**Table 2:** Factors affecting RDW.

	RDW at birth		RDW DOL 14		RDW DOL 28	
	Univariate analysis	Multivariate analysis	Univariate analysis	Multivariate analysis	Univariate analysis	Multivariate analysis
	*P* value	*P* value	*P* value	*P* value	*P* value	*P* value
Gestational age^a^	0.04	0.008	< 0.001	0.76	< 0.001	0.80
Birth weight^a^	< 0.001	0.002	< 0.001	0.13	< 0.001	0.06
Male phenotype^b^	0.44	–	0.48	–	0.18	–
CAM^b^	< 0.001	0.03	0.22	–	0.36	–
Antenatal steroid^b^	0.38	–	0.47	–	0.82	–
PROM^b^	0.08	–	0.17	–	0.49	–
HDP^b^	< 0.001	0.03	0.99	–	0.22	–
RDS^b^	0.87	–	0.67	–	0.11	–
SGA^b^	< 0.001	0.02	0.03	0.03	0.001	0.33
PDA^b^	0.43	–	0.86	–	0.56	–
BPD^b^	0.08	–	< 0.001	0.005	< 0.001	< 0.001
Duration of oxygen supplementation^a^	0.35	–	0.11		0.002	0.84
Nitric oxide therapy^b^	0.14	–	0.008	0.23	0.002	0.47
RBC transfusion before DOL 14^b^	0.92		< 0.001	< 0.001	0.004	0.02
Number of RBC transfusion during neonatal period^a^	0.67	–	< 0.001	0.40	0.004	0.56
Apgar score at 1 min^a^	0.65	–	0.001	0.20	0.003	0.54
Apgar score at 5 min^a^	0.65	–	0.01	0.74	0.06	–
Hemoglobin at birth^a^	0.008	0.99	< 0.001	0.74	< 0.001	0.68
Hemoglobin at DOL 28^a^	–	–	–	**–**	< 0.001	0.14

*CAM* chorioamnionitis, *PROM* premature rupture of membranes, *HDP* hypertensive disorders of pregnancy, *SGA* small for gestational age, *RDS* respiratory distress syndrome, *PDA* patent ductus arteriosus, *BPD* bronchopulmonary dysplasia, *RBC* red blood cell, *DOL* days of life. Multivariate analysis was performed using multiple regression analysis.

^a^Univariate analysis was performed using Pearson’s correlation test.

^b^Student’s t-test.

**Table 3:** Characteristics of BPD and non-BPD infants.

	BPD (N = 85)	Non-BPD (N = 91)	Univariate analysis	Multivariate analysis	
			*P* value	*P* value	95% CI
Gestational age, mean ± SD, week	24.9 ± 1.6	26.1 ± 1.8	< 0.001^b^	0.32	0.79 (0.51–1.25)
Birth weight, mean ± SD, gram	646 ± 157	937 ± 249	< 0.001^b^	0.005	0.99 (0.99–1.00)
Male phenotype, n (%)	44 (51.8)	43 (47.3)	0.76^a^	–	
CAM, n (%)	43 (50.6)	38 (41.7)	0.13^a^	–	
Antenatal steroid, n (%)	65 (76.5)	71 (78.0)	0.88^a^	–	
PROM, n (%)	19 (22.4)	19 (20.8)	0.45^a^	–	
HDP, n (%)	8 (9.4)	13 (14.3)	0.36^a^	–	
RDS, n (%)	70 (82.3)	75 (82.4)	0.55^a^	–	
SGA, n (%)	17 (20.0)	11 (12.1)	0.23^a^	–	
PDA, n (%)	44 (51.8)	46 (50.5)	0.96^a^	–	
Duration of oxygen supplementation ± SD, day	16.7 ± 8.1	9.9 ± 8.0	< 0.001^b^	0.001	1.16 (1.06–1.27)
Nitric Oxide, n (%)	16 (18.8)	3 (3.3)	0.001^a^	0.11	0.18 (0.02–1.53)
Apgar score at 1 min, median (IQR)	3 (2–5)	4 (2–6)	0.001^c^	0.10	0.68 (0.43–1.08)
Apgar score at 5 min, median (IQR)	6 (3–7)	7 (4–8)	0.005^c^	0.14	1.33 (0.91–1.94)
RBC transfusion before DOL 14, n (%)	33 (38.8)	28 (30.7)	0.27^a^		
Number of RBC transfusions during neonatal period, median (IQR)	1 (0–6)	1 (0–6)	0.016^c^	0.742	0.90 (0.46–1.66)
Hb at birth mean ± SD (g/dL)	14.2 ± 2.3	15.7 ± 2.8	0.001^b^	0.91	0.98 (0.76–1.28)
Hb at DOL 28 mean ± SD (g/dL)	9.8 ± 1.8	10.3 ± 1.7	< 0.001^b^	0.74	0.93 (0.65–1.35)
RDW at birth, mean ± SD (%)	16.4 ± 2.1	15.9 ± 1.3	0.03^b^	0.62	0.89 (0.56–1.41)
RDW DOL 14, mean ± SD (%)	21.1 ± 3.6	17.6 ± 2.5	< 0.001^b^	0.67	1.03 (0.84–1.32)
RDW DOL 28, mean ± SD (%)	22.2 ± 3.2	18.2 ± 2.4	< 0.001^b^	0.001	1.63 (1.22–2.19)

*IQR* interquartile range, *CAM* chorioamnionitis, *PROM* premature rupture of membrane, *HDP* hypertensive disorders of pregnancy, *SGA* small for gestational age, *RDS* respiratory distress syndrome, *PDA* patent ductus arteriosus, *RBC* red blood cell, *Hb* hemoglobin, *RDW* red cell distribution width, *DOL* days of life.

P-value was compared between BPD and non-BPD neonates.

^a^Chi-square test.

^b^Student’s t test.

^c^Wilcoxon rank sum test.

Next, we investigated the association between RDW at DOL 28 and BPD in infants who did not receive RBC transfusion (n = 74). As shown in supplemental Figure S2, RDW at DOL 28 in BPD infants who did not receive RBC was also significantly higher than that in non-BPD infants who did not receive RBC.

### Utility of predictors for assessing the onset and severity of BPD in premature infants

**Figure 4:**
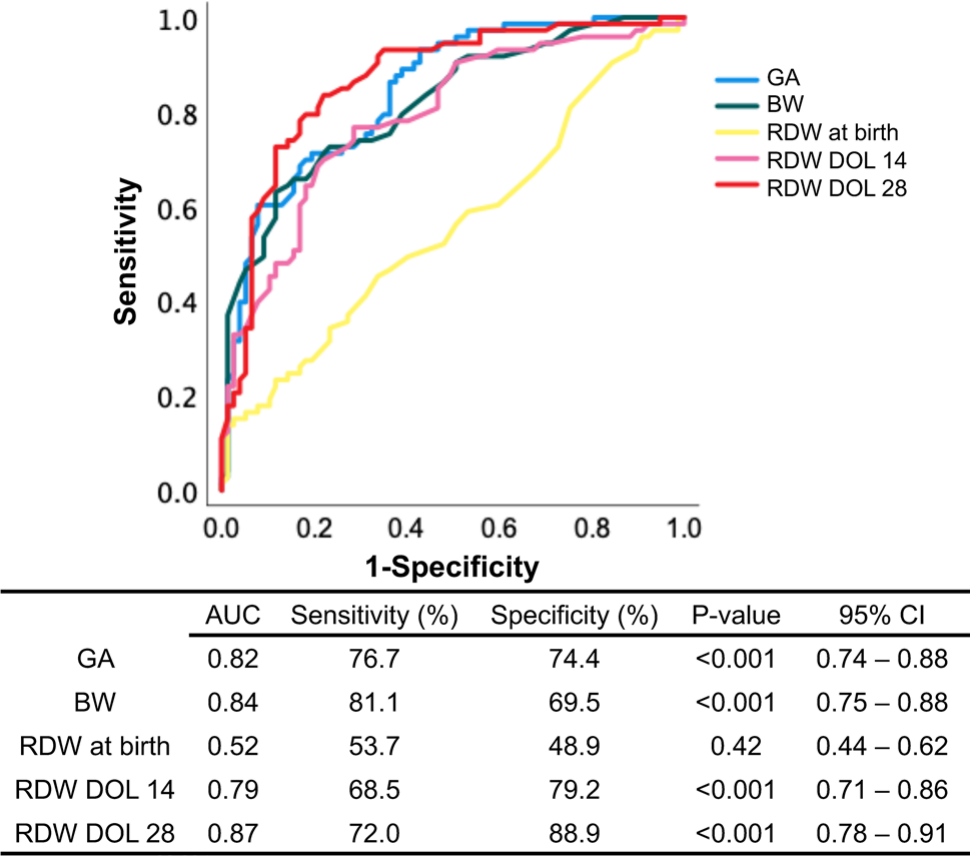
Comparison of receiver operating characteristics by red cell distribution width (RDW), birth weight, and gestational age to distinguish infants with and without BPD. Blood samples at birth and DOL 28 (28 days of life) were obtained from all subjects. Blood samples at DOL 14 were obtained from 152 infants. *BPD* bronchopulmonary dysplasia, *DOL* days of life, *GA* gestational age, *BW* birth weight, *AUC* area under the curve, *CI* confidence intervals.

As shown in Fig. 4, ROC curves were analyzed to determine the utility of predictors to assess the onset of BPD in premature infants. Among the predictors, gestational age (AUC 0.82, 95% CI 0.74–0.88, *P* < 0.001), of birth weight (AUC 0.84, 95% CI 0.75–0.88, *P* < 0.001) and RDW at DOL 28 (AUC 0.87, 95% CI 0.78–0.91, *P* < 0.001), receiver operating characteristic analysis for RDW in infants with and without BPD revealed that RDW at DOL 28 detected BPD with a high area under the curve. A threshold of RDW > 22.5% at DOL 28 identified BPD with 72.0% sensitivity and 88.9% specificity, *P* < 0.001 (Fig. 4).

**Figure 5:**
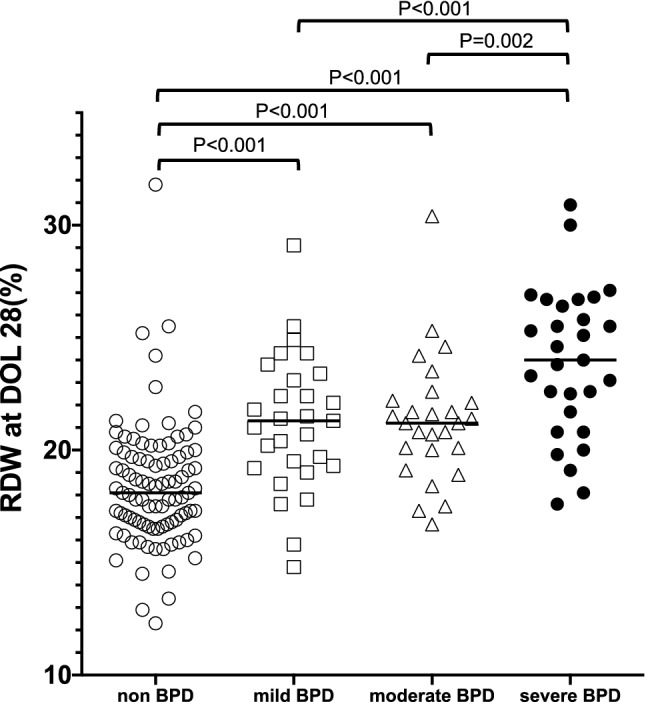
The correlation between red cell distribution width (RDW) at DOL 28 and severity of BPD (non-BPD: n = 91, mild BPD: n = 29, moderate BPD: n = 27, severe BPD: n = 29). The *x*-axis represents severity of BPD, and *y*-axis is RDW (%). Horizontal bars denote the median in each group of infants. Differences in RDW at DOL 28 among non-BPD, mild-BPD, and moderate-severe BPD were evaluated by Kruskal–Wallis followed by Dunn’s Bonferroni post hoc test for multiple comparison. *BPD* bronchopulmonary dysplasia, *DOL* days of life.

Next, we evaluated the relationships between median RDW at DOL 28 and severity of BPD (Fig. 5). RDW at DOL 28 with mild BPD (18.1% vs. 21.3%, *P* < 0.001), moderate BPD (18.1% vs. 21.2%, *P* < 0.001), and severe BPD (18.1% vs. 24.0%, *P* < 0.001) were significantly higher than those with non-BPD, respectively. Furthermore, there are significant differences of RDW at DOL 28 among mild, moderate and severe BPD cohorts (Fig. 5).

## Discussion

To the best of knowledge, this retrospective study is one of only a few studies investigating the relationship between RDW in the postnatal period and BPD. Although Garofoli et al. previously suggested that RDW in the first month of life among BPD infants was higher than that in non-BPD infants^13^, the sample size of preterm infants in their reports was small (n = 41). Furthermore, they did not investigate the associations between RDW and severity of BPD. This study revealed that RDW at DOL 28 in preterm infants born at less than 30 weeks’ gestational age is an independent risk factors for BPD and severity of BPD in a large cohort. This result is consistent with accumulating data showing that RDW is important in the pathogenesis of many disease including lung disease.

The mechanism by which RDW is prognostic for many diseases is unclear. RDW shows the variability of circulating red blood cell size, which is a parameter reflecting the heterogeneity of red blood cell volume. Early RDW was typically used in the diagnosis of anemia, but RDW has been known to be an independent predictor of morbidity and mortality in adult patients with heart disease and lung disease such as chronic heart failure, coronary artery diseases, pulmonary artery hypertension, obstructive sleep apnea syndrome, and chronic obstructive pulmonary disease^8–11^.

The clinical sequelae of BPD include chronic inflammation and intermittent hypoxia. In particular, some previous studies reported that the severity of BPD is associated with the severity of chronic hypoxia and inflammation. Chronic intermittent hypoxia is positively associated with elevated RDW, via intermittent surges of erythropoietin^14^. This study also suggested that RDW at DOL 28 in premature infants with prolonged oxygen supplementation was significantly higher than those without. On the other hand, some suggest that inflammation, a key factor in many chronic diseases, may specifically affect erythropoiesis leading to increased RDW. Lippi et al. showed that RDW might be related to chronic inflammation, due to inflammatory cytokines inhibiting the proliferation of erythroid progenitor cells by inhibiting endothelial nitric oxide production, which was considered to be a factor stimulating erythroid progenitor cell proliferation that affects RDW^15^. To date, many studies suggested that inflammatory cytokines and oxidative stress are associated with the pathogenesis of BPD^16–18^.

Another possible explanation for increased RDW is that oxidative stress negatively impacts red cell survival leading to increased RDW. Approximately 16–25% of BPD patients will develop pulmonary hypertension (PH)^19–21^, which is one of the leading causes of death in these circumstances. In this study, among moderate/severe BPD patients, 19% of infants were treated with pulmonary vasodilator medications including iNO for BPD-PH. Regarding adult patients with PH, many studies reported that high RDW was elevated in PH^9,22^. Because of their incomplete lung development, infants with moderate/severe BPD and PH are exposed to chronic or intermittent hypoxia. In this study, duration of oxygen supplementation in BPD infants was significantly higher than in non-BPD infants. Endothelial cells and the alveolar type II cells are especially susceptible to oxidative stress^5^. As for the pathogenesis of BPD, endothelial cell function is crucial for angiogenesis and alveolarization in the neonatal lung^23^. A previous study suggested that elevated RDW may be caused by endothelial dysfunction and associated with decreased flow-mediated dilation^24^. Thus, we speculate that elevated RDW might reflect the lung endothelial dysfunction in BPD infants.

We also observed an association between RDW and perinatal factors in preterm infants. Although SGA and GA were associated with RDW at birth, SGA and BPD were significantly associated with RDW at DOL 14. On the other hand, BPD and red blood cell (RBC) transfusion were significantly associated with RDW at DOL 28. Furthermore, we found that mean RDW at birth in premature infants born at < 30 weeks was higher than those in children and adults. In terms of a reference interval for RDW, some studies have been informative. A previous study suggested that mean RDW at birth in premature infants born at < 30 weeks was 17.6%^25^. However, they did not investigate the correlations between gestational age, birth weight, and RDW at birth in premature infants born at < 30 weeks. Interestingly, we found that RDW at birth was negatively correlated with birth weight, however, it was weakly correlated with gestational age. Furthermore, the present study also suggested that RDW on DOL 14 and 28 were not associated with birth weight and gestational age in multivariate analysis. We speculate that RDW at birth might be affected by how erythropoiesis is influenced by birth weight, but mechanisms remain to be further elucidated.

Alur et al. suggested that race affected red blood cell parameters in very low birth weight neonates^26^. On the other hand, Christensen et al. reported that a 15.5–20% RDW reference interval at birth did not change over the first two weeks except for those receiving RBC transfusion^27^. They also suggested that preterm neonates had a higher upper reference limit associated with RBC transfusions^27^. Spadaro et al. reported that RBC transfusion increased RDW in intensive care unit patients^28^. This study also suggests that RBC transfusion before DOL 14 was associated with elevated RDW at DOL 14 and 28.

Our study has several limitations. First, it was performed at a single center with a small cohort of moderate/severe BPD patients. We encountered cases with missing data due to our retrospective design, but all patients included had an RDW measured and were characterized in detail. To further validate our observations, a larger sample size with multiple centers and different ethnic cohorts would be invaluable. Second, we could not investigate the association between serum cytokines and RDW. In other reports of BPD, serum cytokines such as IL-6, TGF-β, and IL-8 were significantly increased^29^. RDW has been known to be altered in states of inflammation^30^. Third, we could not investigate the relationship between brain natriuretic peptide level (BNP) and RDW. Previous studies suggested that RDW is associated with brain natriuretic peptide level (BNP)^31,32^. BNP is also known to be associated with severity of BPD^33–35^. Fourth, we could not investigate correlations between iron supplementation and thalassemia status with RDW. Although other cohorts found that some factors such as iron supplementation and thalassemia status were associated with RDW^36,37^, our Japanese cohort did not include infants with thalassemia, and all infants received iron supplementation. Lastly, in this study, we did not investigate the correlation between RDW and lung function. RDW is reported to reflect lung function in adults^12^. There have been some reports of follow-up lung function tests of BPD infants showing these children continue to have abnormal baseline spirometry with significant airway obstruction^38,39^. Furthermore, a previous study also reported that premature infants with BPD before hospital discharge have decreased lung function compared to healthy infants^40^.

In summary, we conclude that RDW at DOL 28 could serve as a biomarker for predicting BPD, and as a predictor for its severity. The mechanism by which RDW at DOL 28 is associated with the pathogenesis of BPD needs further elucidation.

## Materials and methods

### Ethics approval and compliance

The retrospective single-center study was carried out at Fukushima Medical University Hospital’s Department of Pediatrics NICU (neonatal intensive care unit: NICU) from January 2010 to September 2020. The Ethics Committee of Fukushima Medical University, guided by local policy, national law, and the World Medical Association Declaration of Helsinki, approved this study without requiring informed consent from guardians, but consent could be rescinded in the form of opt-out.

### NICU Patients

In this study, newborns born at less than 30 weeks’ gestational age and in the care of the NICU at Fukushima Medical University any time between January 2010 and September 2020 were included. Newborns with congenital anomalies and missing data for RDW at birth and day of life (DOL) 28 or those who died or transferred prior to postnatal DOL 28 were excluded. Data for analysis included gestational age (GA), phenotypic sex, body weight at birth (BW), inhaled nitric oxide (iNO) in the first 28 days, supplemental oxygen at DOL 28, respiratory distress syndrome (RDS), being small for gestational age (SGA), Apgar scores, and the following maternal complications: chorioamnionitis (CAM), premature rupture of membranes (PROM), and hypertensive disorders of pregnancy (HDP). BPD was defined in accordance with the National Institutes of Health consensus definition for infants^41^. At a postmenstrual age of 36 weeks, the infants were classified into the following groups: mild BPD, defined as the need for supplemental oxygen at ≥ 28 days but not at 36 weeks’ postmenstrual age; moderate BPD was defined as the need for supplemental oxygen at 28 days, in addition to supplemental oxygen at FiO_2_ (fraction of inspired oxygen) ≤ 0.30 at 36 weeks’ postmenstrual age; and criteria for severe BPD, which includes the need for supplemental oxygen at 28 days and at 36 weeks’ postmenstrual age, along with mechanical ventilation and/or FiO_2_ > 0.30^42^. SGA was defined as a birth weight of below 1.5 standard deviations that was corrected for the gestational age and sex in accordance with the criteria from prior work^43^. In this study, we included premature infants treated with iNO in the first 28 days for hypoxic respiratory failure (defined needing mechanical ventilation with an oxygenation index [OI] ≥ 10) or pulmonary hypertension identified by echocardiogram^44^. The indications for red blood cell transfusion in NICU were hemoglobin < 7 g/dL, hemoglobin < 11 g/dL with oxygen supplementation, or hemoglobin < 12 g/dL with infants within 24 h of birth.

### RDW and hemoglobin measurements

Blood samples were taken from premature infants at birth, 14 and 28 days of life (DOL 14 and DOL 28), respectively. Two hundred fifty microliters of blood from a peripheral vein were collected into a K3 EDTA tube. Complete blood counts were measured using a Sysmex XE-5000 coagulation analyzer (Sysmex, Kobe, Japan) on admission.

### Statistical analysis

Maternal factors and infant characteristics were summarized with descriptive statistics. Data are presented as mean and standard deviation (SD) for normally distributed data, and median with intra-quartile range (IQR) for non-normal data. Student’s t-test was used for comparison of means and Wilcoxon rank sum test for comparison of medians. The chi-square test was used for categorical variables. The Kolmogorov–Smirnov test was used to judge the normality of distribution of serum RDW at birth, DOL 14, and DOL 28. To evaluate the correlation between two parameters, Pearson’s correlation test was calculated. We performed multiple logistic analyses to determine factors significantly associated with BPD as RDW at DOL 28, BW, GA, CAM, HDP, BPD, duration of oxygen supplementation, RBC transfusion before DOL 14, number of RBC transfusion during the neonatal period, and Apgar scores at 1 min and 5 min. Differences in RDW among time points were evaluated by related-samples Friedman's two-way analysis of variance by ranks. Differences in RDW at DOL 28 among non-BPD, mild BPD, moderate BPD and severe BPD were evaluated by Kruskal–Wallis followed by Dunn’s Bonferroni post hoc test for multiple comparison. ROC curve analysis was performed to determine the utility of biomarkers in detecting the presence of BPD. Significance was set at 0.05 (*P* < 0.05). Data analysis was performed with SPSS (version 20.0) and GraphPad Prism version 8 software.

## Supplementary Information

Supplementary Information 1.

Supplementary Figure S1.

Supplementary Figure S2.

## Data Availability

The datasets analyzed during the current study are not publicly available, because the consent obtained from the participants specified that the data can be used only for research purposes at our institution. The datasets can only be available from the corresponding author upon reasonable request and after the approval of the ethics committee of Fukushima Medical University.
