# Author Correction: Red cell distribution width as a predictor for bronchopulmonary dysplasia in premature infants

**DOI:** 10.1038/s41598-021-93373-8

**Published:** 2021-07-05

**Authors:** Hayato Go, Hitoshi Ohto, Kenneth E. Nollet, Kenichi Sato, Hirotaka Ichikawa, Yohei Kume, Yuji Kanai, Hajime Maeda, Nozomi Kashiwabara, Kei Ogasawara, Maki Sato, Koichi Hashimoto, Mitsuaki Hosoya

**Affiliations:** 1 Department of Pediatrics, Fukushima Medical University School of Medicine, Hikarigaoka 1, Fukushima, Japan; 2 Fukushima Medical University, Fukushima, Japan; 3 Department of Blood Transfusion and Transplantation Immunology, Fukushima Medical University School of Medicine, Fukushima, Japan

Correction to: *Scientific Reports* https://doi.org/10.1038/s41598-021-86752-8, published online 31 March 2021

The original version of this Article contained errors in the reported values of red cell distribution widths at day of life 28, in which some mean values were reported where median values were intended. The authors have recalculated all values as a precaution.

As a result, the numbers in the Article were corrected as follows.

In the Abstract,

“RDW at DOL 28 with mild BPD (18.3% vs. 21.2%, P < 0.001), moderate BPD (18.3% vs. 21.2%, P < 0.001), and severe BPD (18.3% vs. 23.9%, P < 0.001) were significantly higher than those with non-BPD, respectively.”

now reads:

“RDW at DOL 28 with mild BPD (18.1% vs. 21.3%, P < 0.001), moderate BPD (18.1% vs. 21.2%, P < 0.001), and severe BPD (18.1% vs. 24.0%, P < 0.001) were significantly higher than those with non-BPD, respectively.”

In the Results section, under the subheading ‘Utility of predictors for assessing the onset and severity of BPD in premature infants’,

“RDW at DOL 28 with mild BPD (18.3% vs. 21.2%, P < 0.001), moderate BPD (18.3% vs. 21.2%, P < 0.001), and severe BPD (18.3% vs. 23.9%, P < 0.001) were significantly higher than those with non-BPD, respectively.”

now reads:

“RDW at DOL 28 with mild BPD (18.1% vs. 21.3%, P < 0.001), moderate BPD (18.1% vs. 21.2%, P < 0.001), and severe BPD (18.1% vs. 24.0%, P < 0.001) were significantly higher than those with non-BPD, respectively.”

Additionally, the text of the original Article was revised for clarity as follows.

In the Abstract,

“Red blood cell distribution width (RDW), a measure of the variation red blood cell size, could reflect oxidative stress and chronic inflammation in many diseases such as cardiovascular, pulmonary, and other diseases.”

now reads:

“Red blood cell distribution width (RDW), a measure of the variation of red blood cell size, could reflect oxidative stress and chronic inflammation in many diseases such as cardiovascular, pulmonary, and other diseases.”

“Compared with non-BPD infants, mean RDW at birth DOL 14 (21.1% vs. 17.6%, P < 0.001) and DOL 28 (22.2% vs. 18.2%, P < 0.001) were significantly higher in BPD infants.”

now reads:

“Compared with non-BPD infants, mean RDW at DOL 14 (21.1% vs. 17.6%, *P* < 0.001) and DOL 28 (22.2% vs. 18.2%, *P* < 0.001) were significantly higher in BPD infants.”

“Furthermore, there are significant differences of RDW at DOL 28 between mild, moderate, and severe BPD.”

now reads:

“Furthermore, there are significant differences of RDW at DOL 28 among mild, moderate, and severe BPD.”

In the legend of Table 1,

“*PROM* premature rupture of membrane”

now reads:

“*PROM* premature rupture of membranes”

In the legend of Table 2,

“*PROM* premature rupture of membrane”

now reads:

“*PROM* premature rupture of membranes”

In the Results section, under the subheading ‘Clinical characteristics and RDW in preterm infants’,

“Multivariate analysis revealed that in BPD infants, duration of oxygen supplementation in BPD infants was significantly longer than in non-BPD infants (P = 0.001, odds ratio 1.16; CI 1.06–1.27). Although RDW at birth and DOL 14 were not significantly higher in BPD infants, RDW at DOL 28 was significantly higher in BPD infants (P = 0.001, odds ratio 1.63; 95% 1.22–2.19) (Table 3).”

now reads:

“Multivariate analysis revealed that in BPD infants, duration of oxygen supplementation in BPD infants was significantly longer than in non-BPD infants (P = 0.001, odds ratio 1.16; 95% CI 1.06–1.27). Although RDW at birth and DOL 14 were not significantly higher in BPD infants, RDW at DOL 28 was significantly higher in BPD infants (P = 0.001, odds ratio 1.63; 95% CI 1.22–2.19) (Table 3).”

Also in the Results section, under the subheading ‘Utility of predictors for assessing the onset and severity of BPD in premature infants’,

“Next, we evaluated the relationships between RDW at DOL 28 and severity of BPD (Fig. 5).”

now reads:

“Next, we evaluated the relationships between median RDW at DOL 28 and severity of BPD (Fig. 5).”

In the Discussion section,

“Although Garofoli et al. previously suggested that RDW at first month of life in BPD infants was higher than that in non-BPD infants^13^, the sample size of preterm infants in their reports was small (n = 41).”

now reads:

“Although Garofoli et al. previously suggested that RDW in the first month of life among BPD infants was higher than that in non-BPD infants^13^, the sample size of preterm infants in their reports was small (n = 41).”

“Interestingly, we found that RDW at birth was negatively correlated with birth weight, however, was weakly correlated with gestational age.”

now reads:

“Interestingly, we found that RDW at birth was negatively correlated with birth weight, however, it was weakly correlated with gestational age.”

“We speculate that RDW at birth might be affected by erythropoiesis dependent on birth weight. The mechanism by which RDW at birth is associated with birth weight remains to be further elucidated.”

now reads:

“We speculate that RDW at birth might be affected by how erythropoiesis is influenced by birth weight, but mechanisms remain to be further elucidated.”

In the Materials and methods section, under the subheading ‘Statistical analysis’,

“Data are presented as median and standard deviation (SD) for normally distributed data, and median with intra-quartile range (IQR) for nonnormal data.”

now reads:

“Data are presented as mean and standard deviation (SD) for normally distributed data, and median with intra-quartile range (IQR) for non-normal data.”

The original Article has been corrected.

