# Supplementary material for: Red cell distribution width as a predictor for bronchopulmonary dysplasia in premature infants
Source: Sci Rep. 2021 Mar 31;11:7221. doi: 10.1038/s41598-021-86752-8 (PMC8012706; doi:10.1038/s41598-021-86752-8)
Supplement: Supplementary file 1 — Supplementary Information 1. [file 41598_2021_86752_MOESM1_ESM.docx]

**Supplemental Figure 1.**

The correlation between gestational age, birth weight and red cell distribution width (RDW) at birth in a sample of 176 premature infants. Pearson’s correlation test was calculated.

**Supplemental Figure 2.**

Red cell distribution width (RDW) in BPD (n=45) and non-BPD infants (n=29) without red blood cell transfusion. Horizontal bars denote the mean in each group of infants. Student’s t-test was used for comparison of means. BPD: bronchopulmonary dysplasia. DOL: days of life.
