# Supplementary figures and images for: Red cell distribution width as a predictor for bronchopulmonary dysplasia in premature infants
Source: Sci Rep. 2021 Mar 31;11:7221. doi: 10.1038/s41598-021-86752-8 (PMC8012706; doi:10.1038/s41598-021-86752-8)

## A

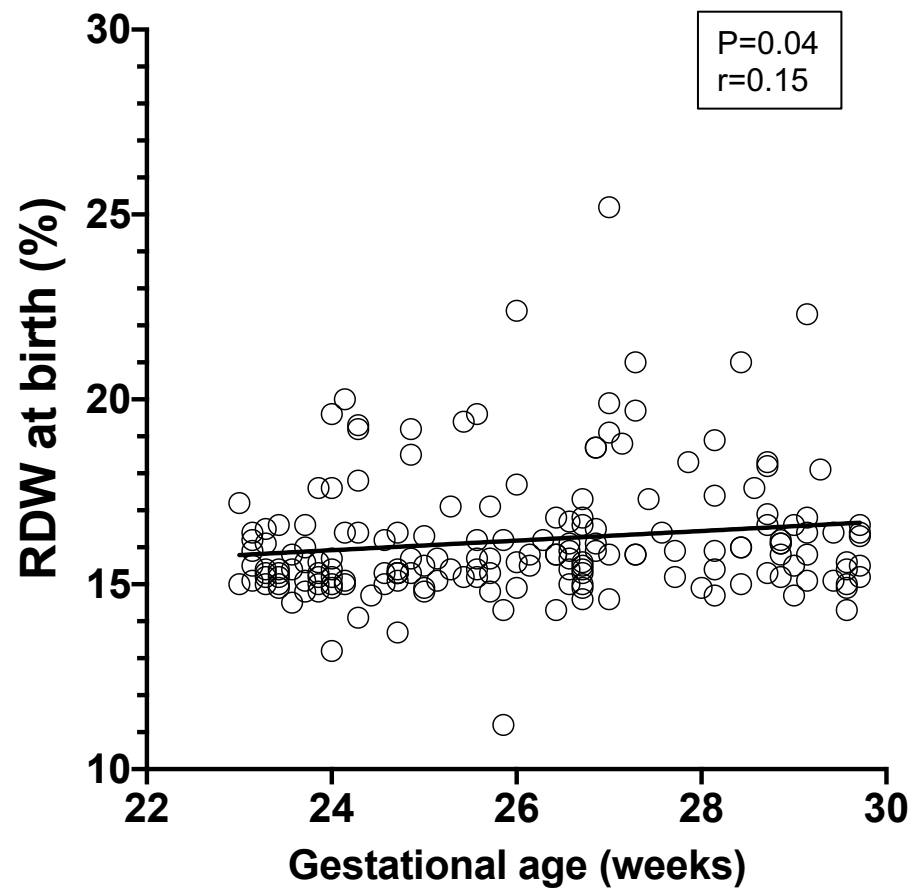

## B

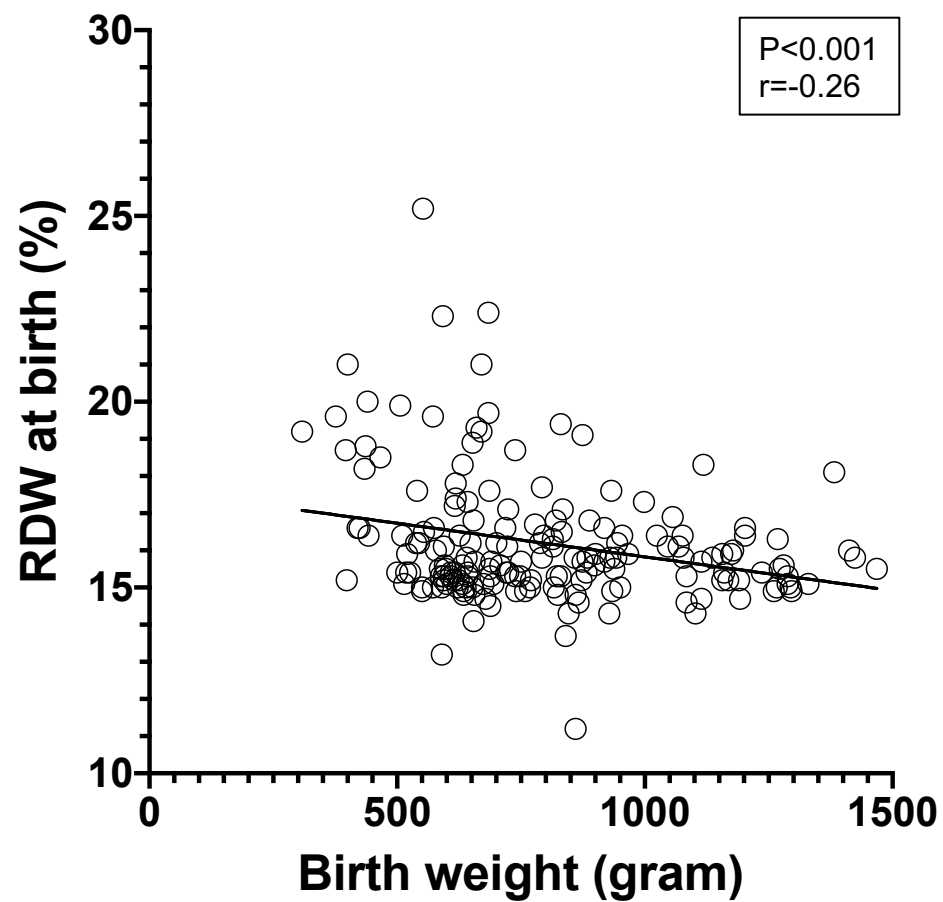

Supplement: Supplementary file 2 — Supplementary Figure S1. [file 41598_2021_86752_MOESM2_ESM.pdf]

**Supplemental Figure S2**

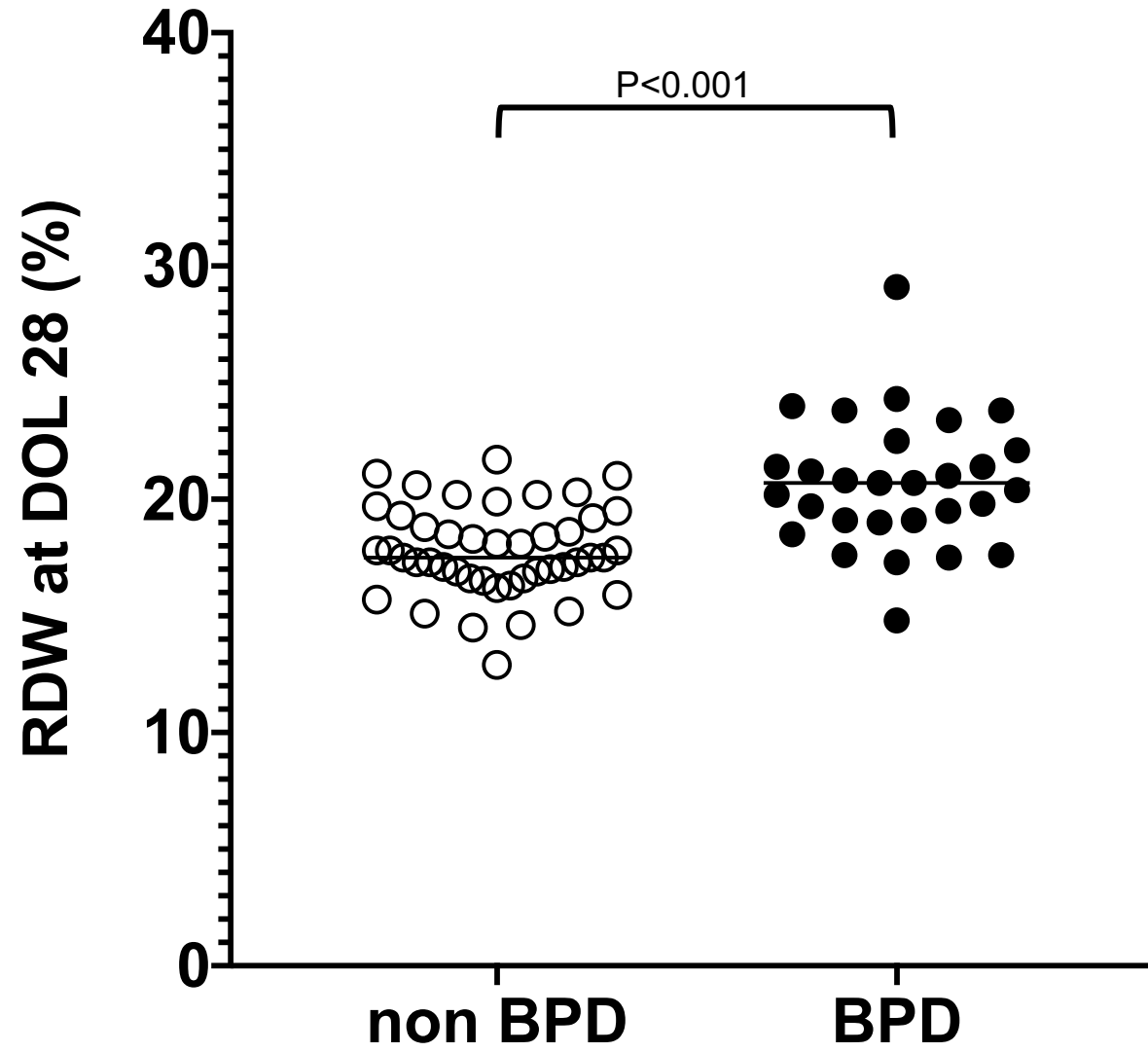

Supplement: Supplementary file 3 — Supplementary Figure S2. [file 41598_2021_86752_MOESM3_ESM.pdf]
